# Menstrual health: a neglected public health problem

**DOI:** 10.1016/j.lana.2022.100399

**Published:** 2022-11-11

**Authors:** The Lancet Regional Health– Americas

Period poverty refers to the lack of access to over-the-counter products to manage menstrual bleeding; however, it can also refer to not having privacy in hygiene management or proper education on menstruation. Globally, more than 500 million women, girls, and others who experience a menstrual cycle do not have suitable access to menstrual hygiene management (MHM); this number, however, is most likely even higher after the COVID-19 pandemic and the current global economic crisis. On average, a person menstruates from age 13 years to 51 years, every 28 days, with a 3–7-day range. This means that they will have 456 periods for about 38 years and use more than 10 000 menstrual products in their lifetime. An increase in price and economic hardship will take a toll on access to these products that should be available for all who menstruate.

In 2019, a global coalition for menstrual health and hygiene was created by the Global Menstrual Collective, calling for global action towards menstrual health and hygiene for all. In the Americas, few countries have taken actions increased access to MHM. In 2021, the government of Mexico passed a law for schools to provide menstrual products for free. In the USA, 23 states and the district of Columbia have tax exemption for menstrual products and, in California, the “Menstrual Equity for All” bill was passed, requiring public schools and colleges to provide menstrual products for free. In 2022, Brazil passed a bill to offer sanitary pads and other menstrual items to low-income students, women who are homeless, and prisoners, as 6 million Brazilians who menstruate have limited access to sanitary products, and 25% of school girls miss school during their period due to lack of access to designated items. However, this is not enough, as access to proper hygiene management and education is still an ongoing issue that access to the products will not solve. Barriers to product access are still very present in the Americas: for example, in 2021, 20% of girls from rural areas of the Dominican Republic missed 2-3 days of school due to a lack of access to menstrual products. In Venezuela, refugees and migrants lack government aid to have the economic resources to buy hygiene products, and a package of tampons can cost up to 3 months’ salary, having a direct impact on the lives of everyone who menstruates.

Even though it is a natural biological process that happens to 1.9 billion people who menstruate, stigma, neglect, and taboos are common in many cultures and countries, and the deconstruction of such issues is a challenge that education and knowledge could help solve together with scientific divulgation. Menstrual health is directly related to proper knowledge of selfcare and the menstrual cycle, and it is related to services of care for the body during menstruation, the diagnosis and treatment of any discomfort, and a respectful environment. However, negative feelings towards menstruation usually start early in children´s lives and can be increased due to the lack of discussion with their family members, resulting in limited information and sometimes misinformation on the topic. Lack of knowledge leads to insecurity and low self-esteem, which can be further exacerbated without access to menstrual products and proper hygiene management.

Menstruation is directly related to human dignity, and deprivation exists when there is no availability of hygiene products, sanitary facilities, and ways to manage menstrual hygiene. Without affordability to purchase menstrual pads, people make use of newspapers, leaves, breadcrumbs, rags, or other objects that could absorb or collect blood, which can increase the risk of urogenital infections. Items such as reusable absorbent pads and menstrual cups could be a sustainable alternative for those who cannot purchase disposable pads. However, cups require sterilisation, and reusable pads need to be fully dried before use to avoid risk of infection, complicating its use in places with high humidity. In places where clean water, electricity, or gas for heat are a challenge, these alternatives are still not ideal. Integrating access to these basic rights can then allow those who menstruate to have a more dignified MHM and access to sustainable and affordable menstrual products.

One of the UN 2030 Sustainable Goals is ending poverty in all forms, and period poverty is included, as it is limited access to basic needs leading to social discrimination and exclusion. All people who menstruate should have access to proper care and basic needs, and menstrual health should be a priority as it is a human right. Poor menstrual hygiene could lead not only to physical health problems such as reproductive and urinary infections, but also to issues that are related to mental health, linking the menstrual cycle to a negative feeling and leaving people who menstruate ashamed. Actions such as providing free access to menstrual products for all who menstruate and public sanitary facilities for proper hygiene are some examples of initiatives that should easily be approved by governments globally. People who menstruate should not have this cost barrier, just as those who do not menstruate. Thus, we urge the Americas to recognise that those who menstruate should not be neglected and act on it as a public health issue.

